# The inhibitory effect of a recent distractor: singleton vs. multiple distractors

**DOI:** 10.1007/s00221-024-06846-3

**Published:** 2024-05-31

**Authors:** Eleanor S. Smith, Trevor J. Crawford

**Affiliations:** Centre for Ageing Research, Department of Psychology, Lancaster University, Lancaster, LA1 4YF England

**Keywords:** Attention, Inhibition, Distractor, Saccadic eye movement, Inhibition of a recent distractor

## Abstract

In the complex interplay between sensory and cognitive processes, the brain must sift through a flood of sensory data to pinpoint relevant signals. This selective mechanism is crucial for the effective control of behaviour, by allowing organisms to focus on important tasks and blocking out distractions. The Inhibition of a Recent Distractor (IRD) Task examines this selection process by exploring how inhibiting distractors influences subsequent eye movements towards an object in the visual environment. In a series of experiments, research by Crawford et al. (2005a) demonstrated a delayed response to a target appearing at the location that was previously occupied by a distractor, demonstrating a legacy inhibition exerted by the distractor on the spatial location of the upcoming target. This study aimed to replicate this effect and to investigate any potential constraints when multiple distractors are presented. Exploring whether the effect is observed in more ecologically relevant scenarios with multiple distractors is crucial for assessing the extent to which it can be applied to a broader range of environments. Experiment 1 successfully replicated the effect, showing a significant IRD effect only with a single distractor. Experiments 2–5 explored a number of possible explanations for this phenomenon.

## Inhibition of visual distractors

Moment-to-moment, our sensory organs are bombarded by competing visual signals as the brain filters the most relevant input to the task at hand from background noise and distractors. To flexibly navigate through complex, natural environments, the ability to sift out visual distractions is critical in attentional control. These competing signals can exert a strong attentional force that pulls our gaze away from the current task and results in slower response times (Theeuwes 1992). The ability to inhibit distracting information and to focus on the task-relevant stimuli is therefore critical for the efficient control of active visual attention. Numerous research studies have suggested that successful navigation of such tasks involves a dual process of directing spatial attention onto the target alongside the inhibition of the distractor (e.g., Wilcockson et al. 2019; Zovko and Kiefer 2013). It has long been argued that the facilitation and inhibition of attention could be two sides of a dual process (Jensen and Mazaheri 2010; Gazzaley and Nobre 2012), however, a rapidly growing body of literature indicates that the suppression of a distractor is not a unitary process but manifests in many forms; which likely reflects multiple underlying neural processes and architectures (Chelazzi et al. 2019; Geng 2014).

Research on the impact of a remote distractor has shown that saccadic eye movements are clearly influenced by visual distractors, affecting both their timing and accuracy (see Walker et al. 1997; McSorley et al. 2012; Benson 2008; Born and Kerzel 2008; Casteau and Vitu 2012). The significance of the relative spatial positioning of the distractor was emphasized in a comprehensive study by Walker et al. (1997), where they differentiated between two types of perturbations: spatial and temporal. Distractors presented within a window of 20 degrees proximal to the target primarily disrupted the amplitude or spatial accuracy of the saccade. These effects on the spatial characteristics of the saccade not only have immediate consequences but can also extend into the future, impacting a saccade several hundred milliseconds after the distractor presentation (Arkesteijn et al. 2018). The trajectory of a saccade is also influenced by the distractor: nearby distractors cause the trajectory to bend towards the target, while distant distractors cause it to bend away from the target (McSorley et al. 2006, 2009). Distractors positioned beyond this 20-degree axis, further from the target, primarily disrupt the timing or latency of the saccade. This timing effect is referred to as the Remote Distractor Effect (Walker et al. 1997).

Several studies have noted a related phenomenon that impacts the distribution of saccadic latencies towards the target. Between 90 and 100 milliseconds (ms) of encountering the target distractor, there is a decline in the dispersion of saccades, commonly referred to as an inhibition effect. The distractor suppresses a proportion of early-onset saccades, leading to a shift in the distribution towards longer latency saccades (Buonocore and McIntosh 2008; Bompas and Sumner 2011). It remains to be determined whether this reflects a single or dual process distinct from the Remote Distractor Effect (Bompas and Sumner 2015). The primary focus of these studies on the Remote Distractor Effect has been on the influence of the distractor on the present saccade. There has been comparatively limited investigation into the enduring influence of the distractor on subsequent saccades. Given the broader importance of inhibitory control in cognitive and neuropsychological investigations, it is crucial to examine the specific characteristics of the inhibitory process that may be essential in preventing inaccurate actions, such as saccadic eye movements.

Explanatory mechanisms of the well-utilised anti-saccade task (AST), has appealed to the concept of inhibitory control together with working memory (Unsworth et al. 2022; Crawford et al. 2011), but there appears to be a clear dissociation in relation to the Inhibition of a Recent Distractor (IRD), a task that is more akin to a spatial negative priming task (Crawford et al. 2005a; Donovan et al. 2012). The AST requires the inhibition of a natural pull towards a sudden target onset, and gaze aversion to the opposite side of the screen (Hallet, 1978). Preparation for the generation of anti-saccade can influence and delay a saccade on the following trial (e.g. Yeung et al. 2014). Although the AST is widely used, it suffers from weak ecological validity since the objective to look away from a salient target without a target to foveate is unusual and counterintuitive. It is far more common, in our everyday lives, to select a target to fixate from a set of non-targets or distractors; for example, when reading a passage of text where a target word is selected form competing words in a sentence. The standard AST does not offer a competing target and therefore requires the ability to disengage attention from a target, as well as the ability to inhibit the distractor. Unlike the AST, the IRD task provides a more realistic measure of inhibitory control by avoiding misleading information about the goal location. In the IRD, the target-distractor relationship is closer to everyday eye movements and visual search tasks. This is perhaps why substantial different success rates for inhibition are reported.

The IRD is a paradigm designed to investigate the characteristics involved in the competitive process used in the selection of a singleton target which is coupled with a distractor (Crawford et al. 2005a; Wilcockson et al. 2019; Polden and Crawford 2021). The first display screen presents, simultaneously, a red target and a green distractor. Participants are asked to fixate on the red target and avoid the green distractor. Here we refer to this as the search display_1_. The second display presents a singleton red target, following a short interval. We refer to this as the display_2_ target. The location of the target in the second display can appear in one of three locations relative to the first display – the same location as the previous target (target-target (T-T), the location of the previous distractor (target-distractor (T-D), or a new location (target-new (T-N). The key finding, revealed by Crawford et al. (2005a) was that the saccadic reaction times to the target in the second display were significantly longer when the target was presented at the location of the previous distractor (T-D), in comparison to the T-T and T-N trials. The inhibition of the distractor in the first search display appeared to be long-lasting (2–5 s) and was detected by its effect on the subsequent saccade in a second target display that was directed to the spatial location of the former distractor. More recent explorations utilising the same paradigm (e.g. Wilcockson et al. 2019; Polden and Crawford 2021), have confirmed the reliability of the effect in both healthy and clinical populations. Crawford, Hill and Higham (2005a) concluded that this saccade slowing was derived from the location of the distractor, rather than another incidental feature of the distractor, such as its colour. However, it is unclear how this effect might apply to different visual contexts. McSorley and Findlay (2003) found that effects on eye-tracking that are observed with single distractors may not necessarily translate to displays with multiple distractors. In the study by Crawford et al. (2005a), the display featured a single distractor. It is crucial to consider how this might translate to more realistic settings where multiple distractors are present, to understand the extent to which the effect can be generalized to a broader array of environments. In another investigation conducted by our team (Donovan et al. 2012), the inhibition of a recent distractor was explored using naturalistic images. Interestingly, no evidence of distractor inhibition was observed in experiments 1–3, although inhibition was detected when a distractor was introduced into the probe display in experiments 4–5. However, it is important to note that the task employed in Donovan et al.’s study involved naturalistic objects, and it remains unclear whether inhibition is present across multiple distractors. Therefore, in the present study, we addressed this issue by utilizing displays identical to those used in the study by Crawford et al. (2005a).

## The present study: what are the effects of multiple distractors on IRD?

Experiment 1 in Crawford et al. (2005a) demonstrated a delayed latency to a target which appeared at the location of a recent distractor, supporting the view that a previous distractor inhibited a saccadic eye movement to a prospective target. This raised the question of whether the presence of multiple distractors would have an equivalent or potentially compound effect? There are several factors that may be peculiar to a display with multiple distractors, compared to a singleton distractor. Various theoretical frameworks feature an attentional selective mechanism where target and non-target items compete for selection (e.g. Humphreys and Duncan 1989; Desimone and Duncan 1995; Findlay and Walker 1999). Thus, mutual inhibitory signals are generated both within and between the display items. The net result may be subject to the weighting and priority assigned to top-down and automatic bottom-up factors. The superior colliculus is the site where critical salience maps for saccadic eye movements are generated (Findlay and Walker 1999; McPeek et al. 2003). When a distractor generates a single peak in the spatiotopic map in the superior colliculus, there is little ambiguity with a winner-takes-all scheme as to the signal that should be inhibited (Findlay and Walker 1999). However, what happens when there is no single “winner” distractor peak, but multiple sites of homogenous activation? Are such sites available for suppression in the same way?

In the Crawford et al. (2005a) study, the display included a single distractor. In the current work we examined the effect with multiple distractors to determine the extent to which the effect might be extrapolated to a wider range of visual contexts. In another study from our group, Donovan et al. (2012) found no evidence of object-based distractor inhibition in experiments 1–3 using multiple distractors, although inhibition was detected when a distracter was introduced into the probe display (experiments 4–5). However, this study (Donovan et al. 2012), used a very different task with naturalistic objects. Therefore, these experiments aimed to answer the question: *Can IRD be observed with multiple distractors, and if so, under what conditions?*

## Experiment 1

### Methods

#### Participants

Twelve participants took part in the experiment (8 female; mean age = 25.7 years; 11 right-handed). All participants had normal or corrected visual acuity (assessed with the Snellen chart), and intact colour vision according to the Ishihara test (Ishihara 1987). No participant had consumed any alcohol in the 12-hours preceding the experiment or taken nicotine in the hour prior to testing. None of the participants had a history of mental health problems and none were currently taking any form of medication. Participants were screened in this manner for all subsequent experiments. This study sample was substantiated by a post-hoc power analysis that established a power of 97% to detect the target effect size, which is above the conventional 80% level. For all power analyses we used G*Power 3.1.9.7 (Faul et al. 2009), and for this design we used the “ANOVA, repeated measures, within factors” procedure. The analysis used alpha = 0.01 and an effect size *f* = 0.65 calculated from the partial η_p_^2^ reported in Experiment 1.

Participant’s eye movements were recorded using the EyeLink desktop 1000, sampling at 500 Hz. The computer monitor size was 24 inches with a resolution of 1366 × 768. Participants were positioned approximately 55 cm from the computer monitor (60 Hz). A chin rest was used to reduce head movements. Participant’s gaze was calibrated prior to the start of the tasks using a 9-point calibration. The stimulus was created and controlled via the use of Experiment Builder Software Version 1.10.1630. The data was analysed and extracted using Data Viewer Software Version 3.2. All described studies were approved by the Lancaster University departmental ethical research committee.

**Fig. 1:**
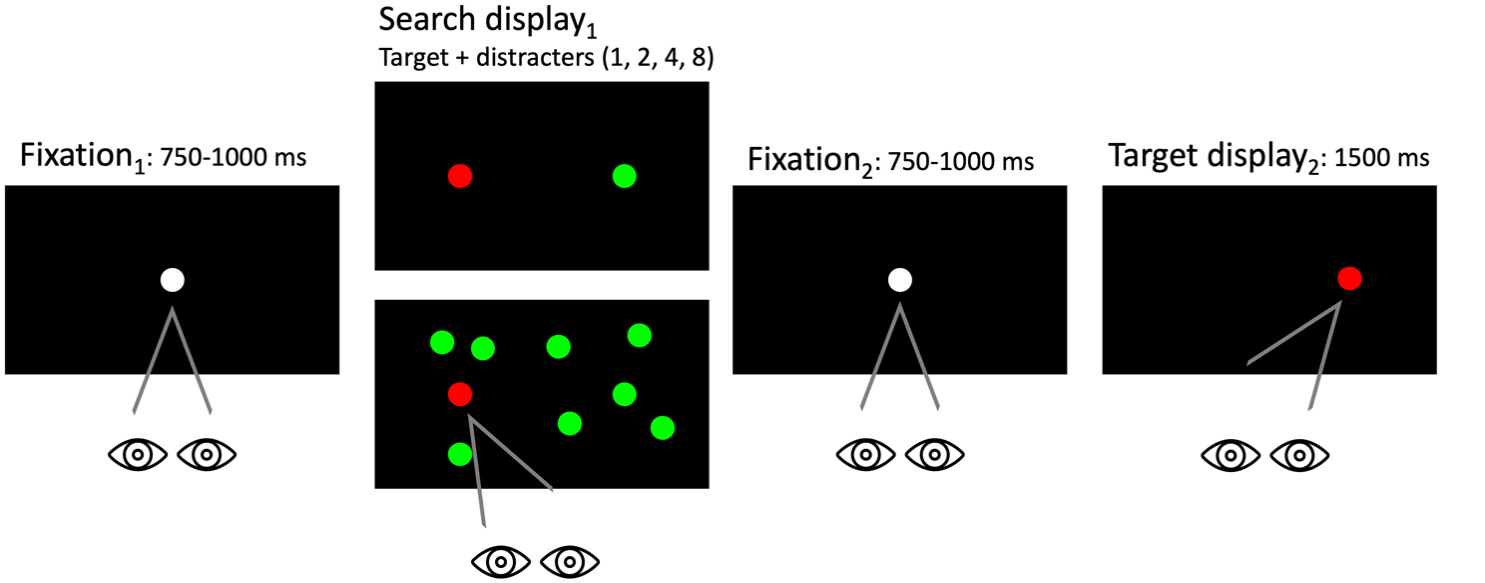
The inhibition of a recent distractor paradigm. Trial began with a white central fixation point for 750–1000 ms, followed by the simultaneous presentation of red and green circular disks for 1500 ms. Participants were expected to look toward the red ‘light’ while ignoring the green distracter. After a randomized fixation period, a single red target was displayed for 1500ms

#### Stimuli and material

4 separate blocks of 1,2,4 or 8 distractors (48 × 4 = 192 trials) were presented using Latin-Square Design. A third of the trials comprised target-target (T-T), target-neutral (T-N) and target-distractor (T-D) trials, randomly interleaved within each block. On a T-T trial the target on display_2_ was presented at the same location of the previously displayed target in display_1_. On the T-N trials the display_2_ target was presented in a new location, not previously occupied by the target or distractor in display_1_. On T-D trials the display_2_ target was presented in the location of one of the previous distractor targets in display_1_. Across all subsequent experiments included in the present research, certain stimuli and procedures remained the same. Where differences occurred, these will be outlined.

#### Task and procedure

Participants were first presented with a white central fixation point for 750–1000 milliseconds (ms), randomised to prevent anticipatory responses (see Fig. 1). After this time elapsed the fixation point was removed and a red and green circular disk (i.e., target distractor display_1_) presented simultaneously for 1500ms. Participants were instructed to look towards the red ‘light’ as quickly and accurately as possible and to ignore the green distractor ‘light’. Search display_1_ was then removed and the central fixation point re-appeared for a randomised interval of 750-1000ms (fixation). Finally, a single red target was displayed for 1500ms (target display_2_). The stimulus onset asynchrony between the search display_1_ and target display_2_ was randomised between 2250-2500ms. A blank interval screen was displayed for 3500ms between trials. The red target was positioned ± 4 ° from the central fixation, either horizontal or vertical locations. Distractors were present along axis 0˚, 45˚, 90˚, 135˚, 180˚, 225˚, 270˚ and 315˚. Targets were present along the principal axis of 0˚, 90˚, 180˚ and 270˚. The fixation point and coloured target measured 15 mm in diameter (visual angle, 1.56°). The mean luminance of the red target was 35.66 lx and the green distractors at 39.57 lx. The experiment was conducted in the eye movement laboratory at Lancaster University. Each participant began with a practice session of 24 trials to familiarise themselves with the conditions of the experiment.

## Results

### Saccade latency: search display_1_

A repeated-measures ANOVA, including the variables distractor set size and trial type for saccadic eye movements to display_1_, demonstrated no significant main effect of trial type (F(2,22) = 0.002, *p* = 0.99, η_p_^2^ = 0.00), or distractor set size (F(3,33) = 1.68, *p* = 0.19, η_p_^2^ = 0.13), and no significant interaction of trial type and distractor set size (F(6,66) = 1.76, *p* = 0.12, η_p_^2^ = 0.14). Therefore, as expected, saccade latency at screen 1 did not differ across the trial types, as the participant experiences the same visual input. For this reason, we will not explore responses towards display_1_ in the further experiments.

### Saccade latency: target display_2_

The critical findings relate to the second display. A repeated-measures ANOVA, including the variables distractor set size and trial type, demonstrated a significant main effect of trial type (F(2,22) = 42.23, *p* < 0.001, η_p_^2^ = 0.79), no main effect of distractor set size (F(3,33) = 0.54, *p* = 0.49), but a significant interaction of trial type condition and distractor set size (F(6,66) = 0.481, *p* < 0.001, η_p_^2^ = 0.30) was present. Post-hoc pairwise comparisons revealed the source of this interaction. Table 1 reveals that for T-D trials there was a selective slowing of saccade reaction times in the single distractor trials in comparison to both T-N (*p* = 0.013) and T-T trials (*p* < 0.001). This effect was not found with a greater number of distractors.

**Table 1:** Saccade latencies for T-T, T-N, and T-D trial types with different distractor set sizes. Note that a significant (slowed latency for T-D trials) is only present when a singleton distractor was presented. * = *p* < 0.001

Distractor Set Size	Mean Saccade Latency (ms) for different trial type conditions		
	T-T	T-N	T-D
1	175	190	206*
2	176	195	200
4	179	189	187
8	182	195	191

* *p* < 0.001

### Saccade error

To determine whether the distinct effect of the single distractor trials can be accounted for by differences in the distribution of errors we compared the proportion of errors as a function of distractor set size. Distractor inhibition errors were defined as those trials in which a saccade was generated towards the distractor, instead of the target in search display_1_. The proportion of error trials for distractor set size (d = 1 (3.73%); d = 2 (2.83%); d = 3 (2.00%); d = 4 (1.99%)) were not significantly different (F(3,33) = 2.12, *p* = 0.117, η_p_^2^ = 0.16) across the display conditions.

### Speed-accuracy trade-off

To explore the possibility of there being a speed-accuracy trade-off in this paradigm, we explored whether a similar effect may be present for the saccade amplitude metric. The results revealed no significant main effect of trial type (F(2,22) = 0.29, *p* = 0.76, η_p_^2^ = 0.03) or distractor set size (F(3,33) = 0.72, *p* = 0.55, η_p_^2^ = 0.06) on saccade amplitude.

### Target-distractor proximity

It was possible that any effects reported in Experiment 1 may have been restricted to targets that were located more distally from the target given that spatial negative priming has been shown to be optimal for more distant distractors (Chao 2011). To determine whether there might be an effect of target proximity in the T-D displays, we separated distractors that were near (90 ˚) and far (180 ˚) from the previous target.

Several analyses were conducted to determine whether near vs. far target-to-distractor visual angles had a clear influence on the level of T-D inhibition. Table 2 shows the mean saccade latencies for T-D trials, at near vs. far locations for each multiple distractor display. These analyses revealed no effects of T-D proximity (near vs. far) (distractor *N* = 1, t(11) = -2.034, *p* = 0.07; distractor *N* = 2 t(11) = 1.97, *p* = 0.08; distractors *N* = 4, t(11) = 0.54, *p* = 0.54; distractor = 8, t(11) =-1.33; *p* = 0.21).

**Table 2:** Saccade latencies for T-D trial types for different set sizes and the location of the target was near vs. far

Proximity	Distractor Set Size	Mean Saccade Latency (ms)
90 ˚		
	1	192.87
	2	204.95
	4	188.10
	8	185.83
180˚		
	1	220.98
	2	196.44
	4	185.25
	8	193.63

### Discussion

Experiment 1 sought to replicate the delay in the initiation of saccadic eye movement observed by Crawford et al. (2005a) when a target appeared in the location of a recent distractor, and to evaluate whether an equivalent effect was evident when multiple distractors are present. With regards to the T-T trial type, responses were significantly faster than that displayed for the T-D and T-N trial types. As such, henceforth we will focus primarily on the T-N and T-D trial types. For T-D trials, there was a selective slowing of saccade reaction times in the single distractor trials when compared to both T-T and T-N trials, although interestingly this effect was not found when more than a single distractor was present. This raises the question, why is it that with multiple distractors we don’t find evidence of saccadic inhibition?

There are several possibilities here, and the subsequent experiments will address these. One possibility is that, because of the multiple distractors presented over wide-ranging visual angles on the screen, their impact on the target may have been diluted. Although the analyses on the effects of T-D proximity appears to undermine this hypothesis we sought further evidence from an alternative approach. Therefore, experiment 2 aimed to explore this idea further by clustering the distractors within a confined region more proximal to the target, to address the possibility that inhibition may be generated more consistently when distractors compete in close proximity to the target.

### What is the effect of spatial clustering on distractor inhibition?

Experiment 1 replicated a previous report of IRD using singleton distractors but failed to detect evidence for IRD with multiple distractors. Note that in comparison to a single distractor condition, the distribution of multiple distractors could vary over a relatively large range of visual angles. Thus, one possibility is that inhibition may be subject to spatial constraints such that inhibition is optimal for distractors within a limited focal spatial region around the distractor. It is plausible that multiple distractors positioned close to the target may be assigned or designated a higher priority within the competitive selection framework (Duncan and Humphreys 1989). Therefore, in experiment 2 we sought to explore whether distractors presented more closely to the target would receive a greater level of inhibition than distractors that were presented further away (Cave and Zimmerman 1997).

## Experiment 2

### Method

#### Participants

Twelve participants took part in the experiment (8 female; mean age = 20.3 years, SD age = 5.4; 10 right-handed).

#### Stimuli and Material

**Fig. 2:**
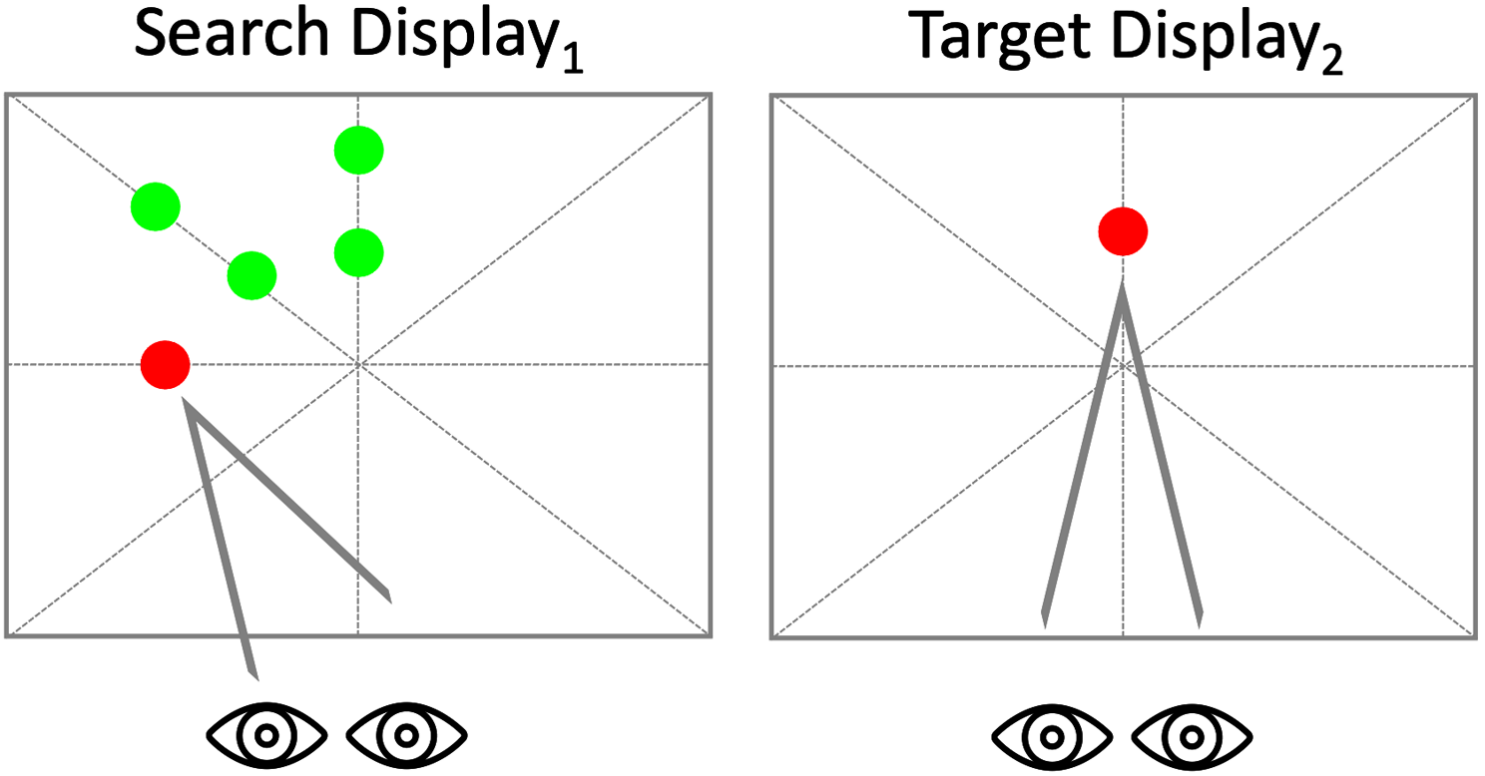
Spatial Clustering adaptation example of Search display_1_ and Target display_2_ of the inhibition of a recent distractor paradigm

Of central interest was the question of whether IRD would be evident when distractors were distributed within a constrained spatial region, therefore the trials consisted of T-D and T-N trials. T-T trials were not included. A new paradigm was run, with the key distinguishing features (from experiment 1) being that the search display_1_ consisted of a red target together with 4 distractors distributed within all within a 45 ˚ sector with targets positioned at 4 ˚ and 8 ˚ (Fig. 2). Set size was not manipulated in this experiment – there were always 4 distractors present, and as in experiment 1 targets were presented randomly at one of the principal axes (0˚, 90˚, 180˚ or 270˚). On T-D trials the target in display_2_ was present at one of the distractor locations in the search display_1_. On T-N trials the target in display_2_ was presented at a new location. The locations of target presentations for T-D and T-N trials were identical. The timing of the T-D and T-N trials were identical to experiment 1. Trials were presented in 3 blocks of 32 trials, yielding a total of 96 trials for each participant.

## Results

### Spatial clustering

To explore the potential influence of the spatial clustering of distractors, paired-samples t-tests were utilised. Mean saccadic reaction times for T-D trials (176ms, SD = 20.5) and T-N trials (174ms, SD = 23.4) were highly similar (t(11) = 0.88, *p* = 0.40). Mean error rates for each condition were not significantly different (t(11) =-0.56, *p* = 0.59), and were also low 0.08% (T-D) and 0.17% (T-N).

### Discussion

Experiment 2 aimed to explore whether restricting multiple distractors within a close confined visual angle of the target would be sufficient to generate inhibition of target by a recent distractor. The spatial clustering of distractors clearly failed to induce inhibition for a multiple distractor set.

### Does distractor inhibition decay over time?

Experiment 1 and 2 confirmed that the spatial distribution of the display did not significantly impact the IRD effect. The following experiment aimed to investigate whether the influence of distractors might be influenced by temporal constraints, related to attention allocation. There is little doubt that perceptual performance is influenced and enhanced by the allocation of spatial attention. The influence of spatial attention on perceptual performance is particularly pronounced in peripheral vision, which is influenced by multiple factors (Kewan-Khalayly et al. 2022). The impact of involuntary attention arises quickly after stimulus onset but diminishes rapidly afterward (Müller and Rabbitt 1989). In contrast, voluntary attention takes longer to initiate (Posner et al. 1982), but is sustained for extended durations (Silver et al. 2007). Research on voluntary attention commonly utilizes an extended interval between the onset of the cue and the stimulus (Posner et al. 1982). Whether or not the attentional mechanism operates in the interval between two displays in the current task is unknown. Therefore, experiment 3 sought to explore whether the limitations on the effect could be due to the temporal factors, such as time intervals, or the stimulus onset asynchrony (SOA) between the two displays. Could it be that with multiple distractors the inhibition is short lived, or that the inhibition requires more time to accumulate? If the brain requires more time to distribute attention, then we would expect to see a larger effect at a long interval (2400 ms) compared to a short interval (400 ms). Alternatively, if the effect is generated rapidly but with a short half-life, then we would expect to see a large effect at the short interval.

## Experiment 3

### Method

#### Participants

Twelve participants took part in the experiment (10 female; mean age = 18.7 years, SD age = 1.2).

#### Stimuli and Material

**Fig. 3:**
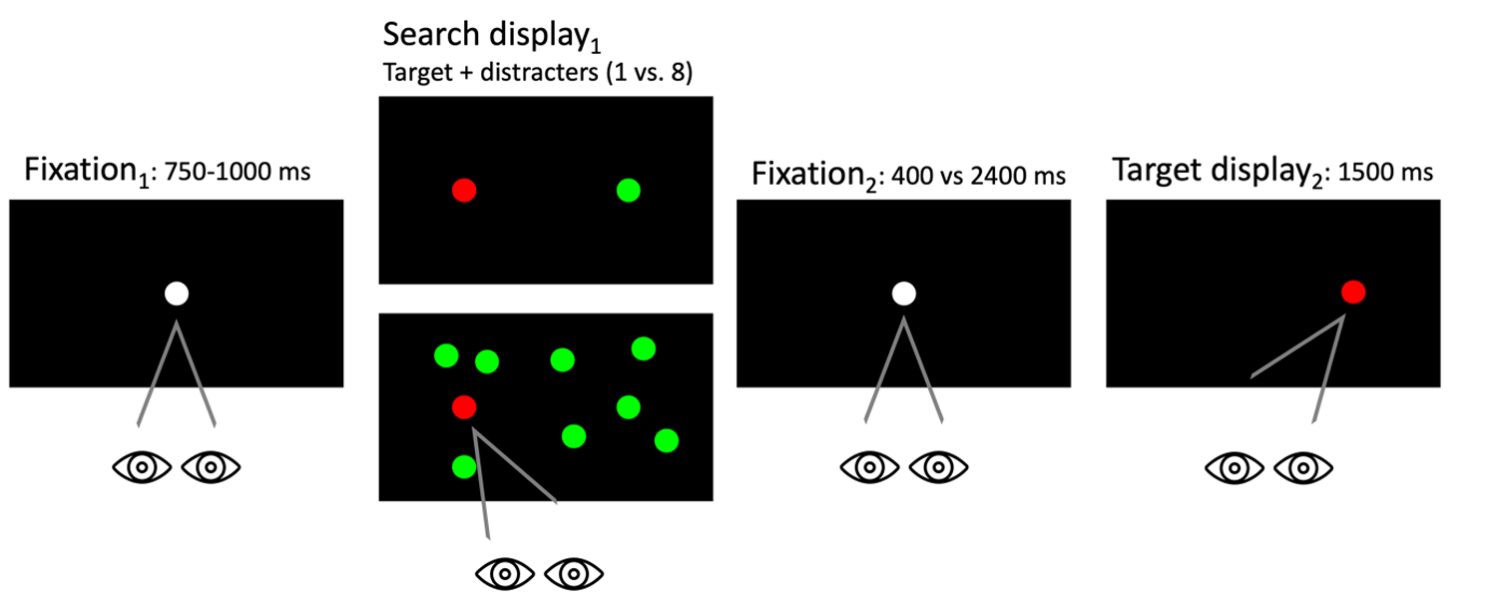
The inhibition of a recent distractor paradigm with differing lengths of time on the fixation_2_ screen, presented for either 400 ms or 2400 ms

The paradigm used was essentially identical to that in experiment 1, with some minor differences. In the search display_1_ either 1 or 8 distractors were presented, as we previously identified no significant differences across other distractor set sizes (2, 4, 8). Additionally, the fixation point was presented for either 400 ms or 2400 ms (differences shown in Fig. 3.) These different stimulus onset asynchronies (SOA) were presented to the participant in separate blocks. Catch trials were randomly distributed to avoid anticipations.

## Results

### Saccade latency: target display_2_

A repeated-measures ANOVA highlighted a significant main effect of trial type (F(1,10) = 3.99, *p* = 0.03, η_p_^2^ = 0.27), but no main effect of SOA (F(1,10) = 2.41, *p* = 0.15, η_p_^2^ = 0.18), or the number of distractors in the set (F(1,10) = 0.53, *p* = 0.48, η_p_^2^ = 0.05). There was a significant trial type by distractor set size interaction (F(2,22) = 17.12, *p* < 0.001, η_p_^2^ = 0.77). Given this significant interaction, the analysis went on to explore how the saccade latency was influenced by the different number of distractors.

### Single distractor

**Fig. 4:**
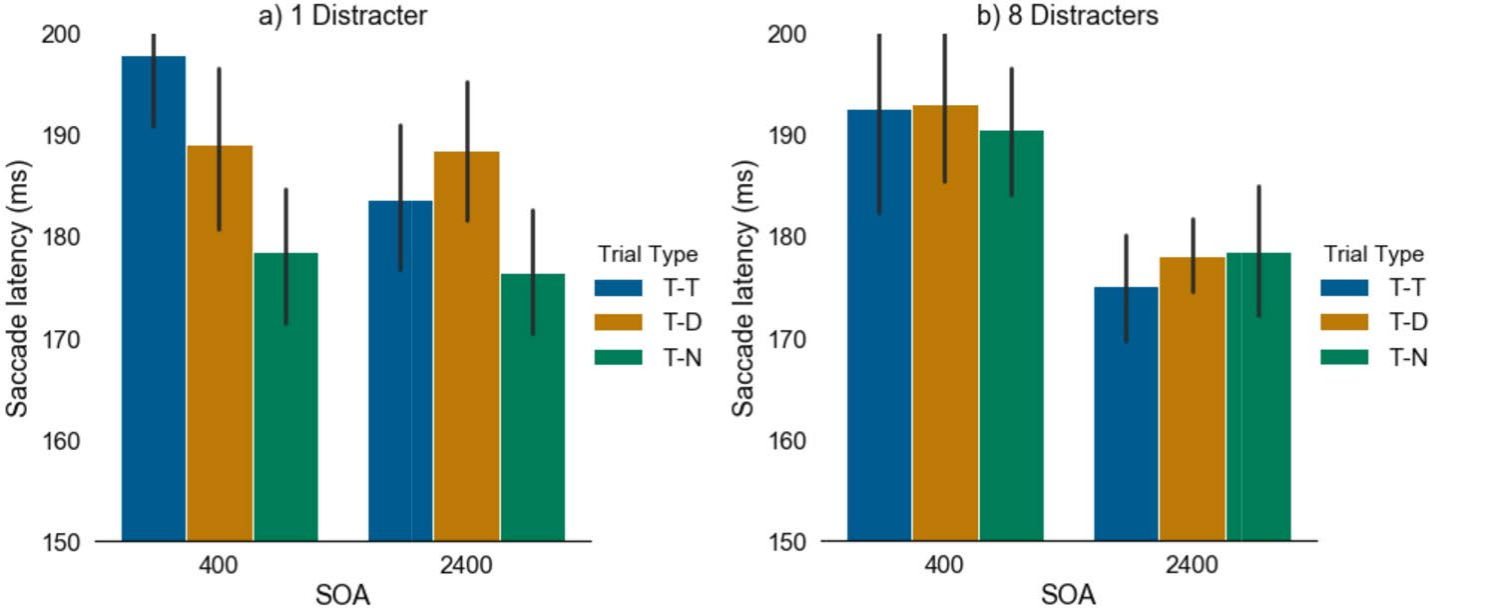
**a**. Saccade latencies across trial types (T-T, T-D, T-N) and SOAs (Stimulus Onset Asynchrony) (400 vs. 2400 ms) when a single distractor was present. **b**. Saccade latencies across trial types (T-T, T-D, T-N) and SOAs (400 vs. 2400 ms) when multiple (8) distractors were present

A repeated-measures ANOVA revealed a significant main effect of trial type (i.e. increased mean latency in T-D trials) when a singleton distractor was presented (T-D mean = 189ms; T-N mean = 177ms; T-T mean = 191ms) (F(1,10) = 6.57, *p* = 0.006, η_p_^2^ = 0.37); but no main effect of SOA (F(1,10) = 0.53, *p* = 0.48, η_p_^2^ = 0.05) or interaction between the two variables (F(1,10) = 2.72, *p* = 0.09, η_p_^2^ = 0.20) (see Fig. 4a).

### Multiple distractors

To explore how the number of distractors may be impacting saccade reaction times, a repeated-measures ANOVA revealed a significant main effect of SOA when multiple (8) distractors were presented (F(1,10) = 5.42, *p* = 0.04, η_p_^2^ = 0.33), but no main effect of trial type (T-D mean = 186ms; T-N mean = 185ms; T-T mean = 184ms) (F(1,10) = 0.08, *p* = 0.92, η_p_^2^ = 0.007) or interaction between the two variables (F(1,10) = 0.23, *p* = 0.80, η_p_^2^ = 0.02) were present. Post-hoc pairwise analyses illustrated that as expected significantly longer reaction times were present for the 400ms SOA compared to the 2400ms SOA (see Fig. 4b).

For a target that was presented with a singleton distractor, there was clear evidence of inhibition at the distractor location for the short and long SOAs. In contrast, for multiple distractors there was no IRD at either SOA.

### Discussion

Experiment 3 sought to explore the effect of time in relation to the IRD effect on a single and multiple distractors. With multiple distractors present, more time may be required for the inhibition to come into play. If this is true, then for multiple distractors inhibition should be detected at the longer (2400 ms) in contrast to the short (400 ms) SOA. Clearly, this was not the case.

Once again, the data replicated the IRD effect, but this was only present with a singleton distractor. In contrast to our hypothesis, the effect disappeared with multiple (8) distractors. Furthermore, there was no significant interaction with SOA either. We can therefore safely rule out the explanation that with longer intervals you are more likely to get inhibition for multiple targets. To summarise the collective findings so far; we report a clear and consistent inhibition for a singleton distractor and the consistent replication of the absence of inhibition with multiple distractors (ranging from 2 to 8 distractor set sizes). There appears to be no clear spatial constraints involved in the lack of the IRD effect (with multiple distractors), whilst there also appears to be no simple temporal constraints that can account for the absence of inhibition for multiple targets.

### Is IRD selective for successful inhibition trials?

Experiment 1–3 explored potential influences of the spatial proximity of distractors in relation to the target and temporal intervals associated with target distractor displays, however, the effect was not significantly altered. This raises the question of the nature and validity of the inhibitory process that we have inferred in the context of target selection against a competing distractor. We therefore decided to return to the dataset collected in experiment 1 to clarify whether successful inhibition in the search distractor display_1_ was critical to the effect. This dataset was utilised as it includes a full range of distractor set sizes (1–8) in the original IRD paradigm, so a detailed comparison could be made on the importance of inhibition in search display_1_.

The original evidence in favour of an active inhibitory process, was derived from the slowing of saccadic reaction times to the distractor location in comparison with target location and new, neutral locations. This interpretation of the IRD effect in terms of an active inhibitory mechanism is compatible with existing theories, such as competitive interactions theory (Theeuwes 1992) and negative priming (Houghton and Tipper 1994). However, the hypothesis of an inhibitory mechanism also makes a key prediction in relation to error and non-error trials. Therefore, the current analysis takes advantage of the fact that a proportion of erroneous saccades were directed towards the distractor, rather than the target in display_1_. Behaviourally these trials can be classified in terms of either successful inhibition (SI) – saccades that were correctly directed to the target, or failed inhibition (FI) – saccades that were initially directed to the distractor (though all saccades were followed by a corrective saccade to the target). Clearly, the presence of inhibition in display_2_ would be weak or absent where inhibition has failed (i.e. FI trials) in display_1_. Therefore, to the extent that IRD is inhibition based, the following predictions follow:

(i) Saccades to the display_2_ target should have faster reaction times following a failure to inhibit a saccade to the distractor than saccades that followed a successful inhibition (FI < SI), and this effect should be specific to T-D trials.
(ii) Saccades to a previously empty location or a location that only contained a target should be unaffected.
(iii) Finally, since there is no evidence for IRD with multiple distractors, this relationship should be specific for the singleton distractor trials.

## Experiment 4

### Method

#### Participants

Twelve participants took part in the experiment (8 female; mean age = 25.7 years; 11 right-handed).

#### Task and Procedure

The same paradigm outlined in Fig. 1 was used.

### Results

**Fig. 5:**
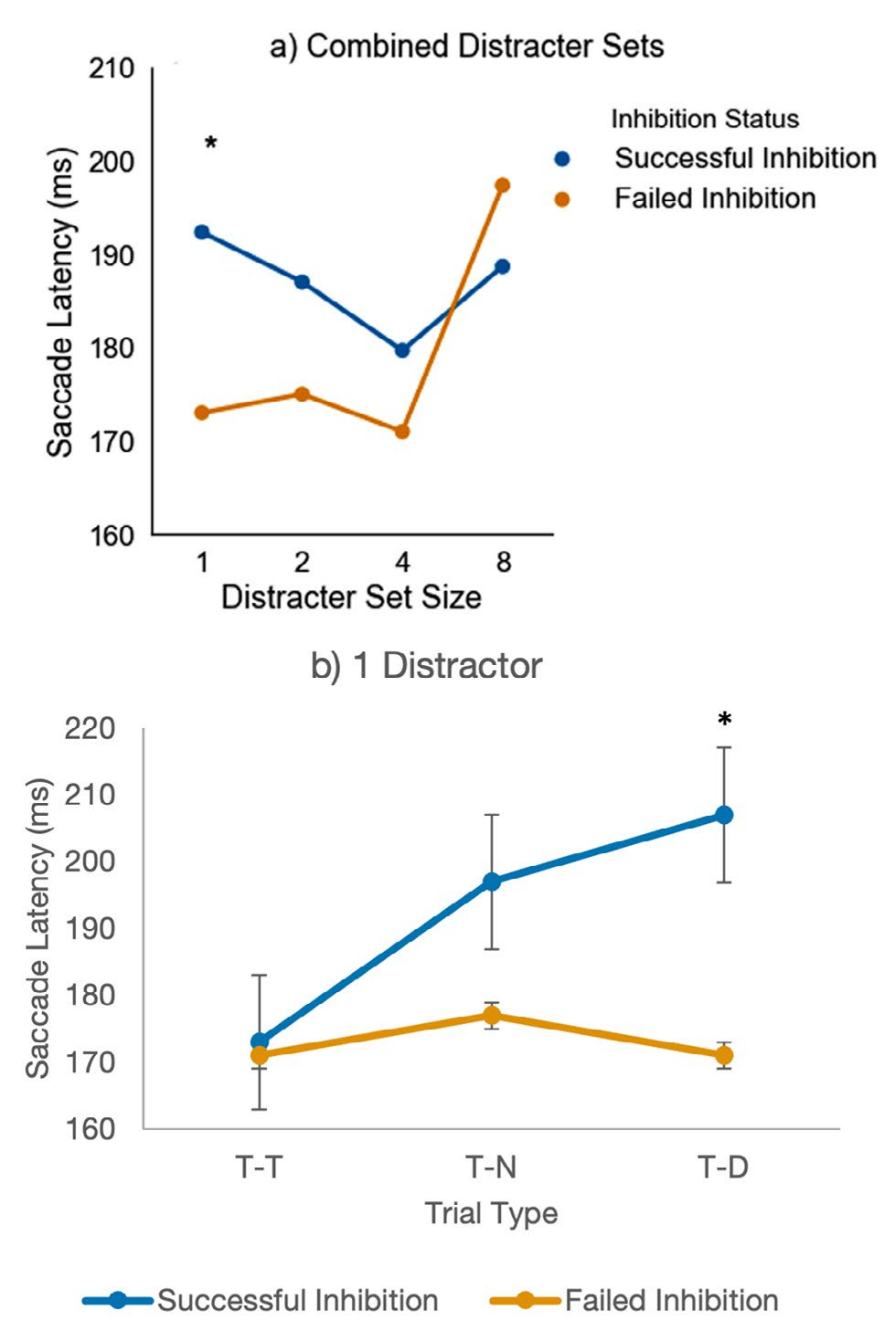
**a**. Saccade latencies across distractor set sizes by inhibition status. Note that a significant difference was found when a single distractor was presented. **b**. Saccade latencies across trial types when a single distractor was present by inhibition status. * = *p* < 0.001. Error bars representing standard error

The first paired samples test compared search display_2_ saccade reaction times on successful inhibition (SI) and failed inhibition (FI) trials for displays (see Fig. 5a and b). Table 3 reveals that saccades were selectively slowed on trials when the distractor was successfully inhibited, but that this pattern was confined to singleton distractors: confirming hypothesis iii. The average saccade latency when a singleton distractor was successfully inhibited was 173 ms in T-T trials, 197 ms in T-N trials, and 207 ms in T-D trials. Hypothesis i was confirmed, with average saccade latencies significantly slowed following a successful inhibition (FI (171 ms) < SI (207 ms)) during T-D trials, and that T-T and T-N trials were unaffected (hypothesis ii).

**Table 3:** Saccade latencies for trials in which inhibition was successful (SI) and inhibition was failed (FI) across T-T, T-N, and T-D trial types. * = *p* < 0.001

Distractor Set	Inhibition Status	Average Saccade Latencies (ms) across conditions		
		T-T	T-N	T-D
1	SI	173	197	{207}*
	FI	171	177	{171}
2	SI	175	187	199
	FI	176	173	176
4	SI	171	176	192
	FI	171	159	183
8	SI	194	183	189
	FI	188	184	220

* TD condition SI vs. FI *p* < 0.001

### Discussion

Experiment 4 sought to compare trials in which there was successful inhibition of distractors in search display_1_ versus trials in which inhibition was unsuccessful (failed inhibition trials). Drawing this comparison is important because evaluating different numbers of distractors allows us to confirm whether there is something unique occurring for displays with a singleton versus multiple distractors. It was predicted that if there was a clear distinction in the type of inhibition taking place, this may be observable when comparing singleton versus multiple distractors, and as such there would be a significant difference between FI and SI for a singleton distractor, compared to multiple (8) distractor trials.

The data demonstrates exactly that. Saccade latencies were prolonged on trials where the distractor was successfully inhibited (SI), whereas on trials where participants couldn’t inhibit the distractor during search display_1_, they had quicker saccade latencies and no Inhibition of a Recent Distractor (IRD). This analysis reveals that there is clear evidence of inhibition taking place for a single distractor trial, which is quite different to the processes taking place for multiple distractors. These results are consistent with the prior experiments findings and provide strong support that active inhibition is limited to a singleton distractor.

### Are eye movements necessary for IRD?

A series of experiments have replicated the original phenomenon of IRD with a single distractor. However, the underlying source of inhibition remains unclear. One possibility is that IRD is generated in response to *competition* between two saccadic programmes: one generated towards the target and the other towards the distractor. Using this conflict in saccadic programming that is created by competition between the pro- and anti- saccade, our previous work (see Crawford et al. 2005a, Experiment 4) suggested that motor competition is not sufficient for the generation of IRD. The critical evidence came from the observation that a prosaccade to the target presented at the location of a previous ‘virtual’ distractor in the AST yielded an essentially identical reaction time to a saccade that was directed towards the previous antisaccade. A single distractor in the absence of a target was not sufficient to generate distractor inhibition. However, it is possible to determine the role of saccadic competition using an alternative approach by simply removing the ‘saccadic target’ altogether. It is possible that inhibition of distractor, in the absence of any target goal, may be a sufficient signal for IRD. In which case IRD should be evident in a “No-go” task in relation to a singleton distractor as revealed in the following experiment. The analyses conducted in experiment 4 above, demonstrated that successful inhibition of a distractor in search display_1_ is a key factor in the IRD effect. If this is the case, it follows that you should also see the effect even if no target is present in search display_1_: a singleton distractor should be sufficient. Experiment 5 sought to explore this hypothesis.

## Experiment 5

### Method

#### Participants

Twelve participants took part in the experiment (10 female; mean age = 18.7 years, SD age = 1.2).

#### Task and Procedure

**Fig. 6:**
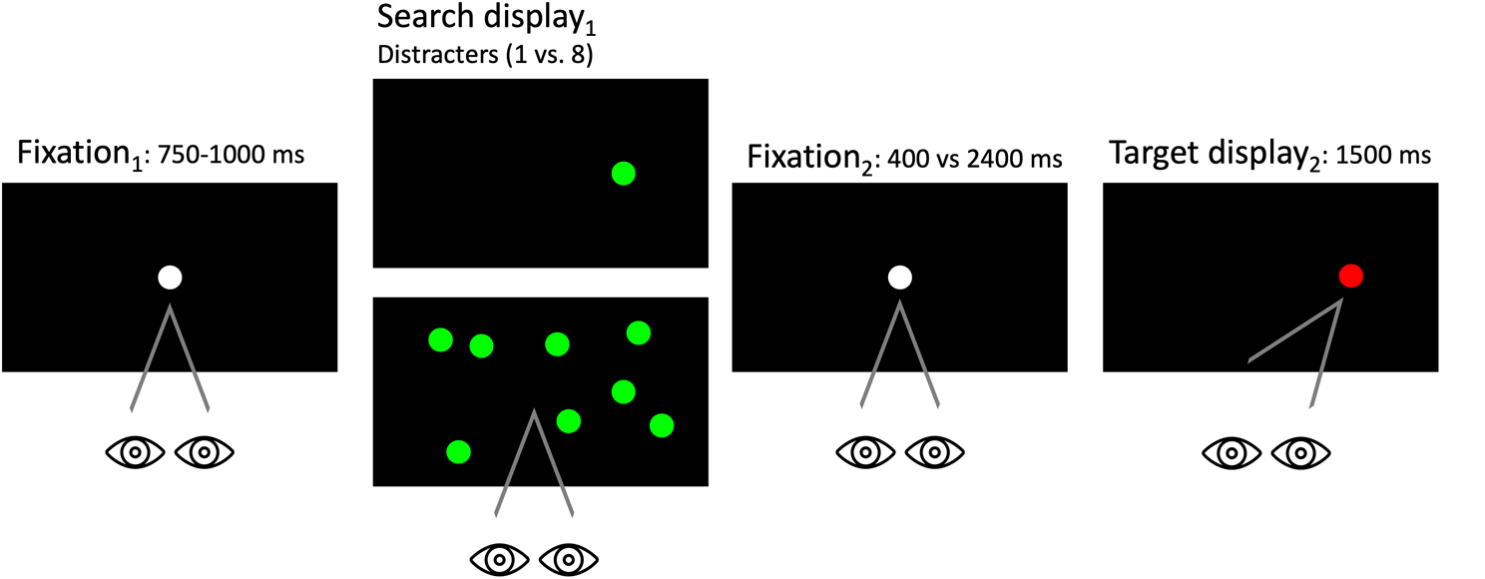
Go/No-go paradigm. Note how for search display_1_ there is no target displayed, only the distractor set. Here, the participant is instructed to maintain fixation centrally, as in fixation_1_

The current experimental stimuli were similar to experiment 3, except for two critical features (see Fig. 6). The search display consisted of either 1 or 8 green distractors. The red saccade target was removed from the search display, with the instruction to ignore all distractors in the initial search display. This yielded a ‘No-go’ task in the first display. Thus, on T-D trials the target in the second display was presented at the location of the distractor in the first no-go display. On T-N trials the target in the second display was presented at new location in relation the previous distractor in the previous ‘No-go’ display. This was followed by a ‘Go’ display consisting of a red target that was presented at the location of either a previous distractor or a new location. There were 2 blocks of 96 trials in each block. Catch-trials were randomly distributed to avoid anticipations. We used display intervals of 400 ms and 2400 ms. If IRD survives the removal of a saccade from the paradigm, this would provide direct support for the role of distractor inhibition.

### Results

**Fig. 7:**
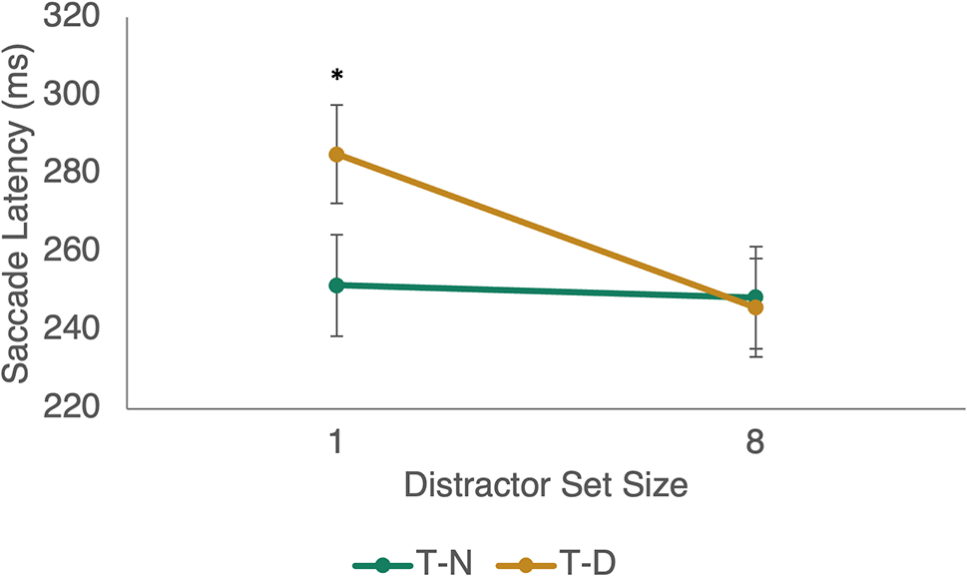
Saccade latencies across distractor set sizes (1, 8) and trial types T-N and T-D. Note how in T-D trials significantly longer latencies were found only when a singleton distractor was present. * = *p* < 0.05. Error bars represent standard error

A repeated-measures ANOVA produced a statistically significant main effect of trial type (T-N vs. T-D) (F(1,10) = 19.13, *p* < 0.001, η_p_^2^ = 0.66) and the distractor set size (F(1,10) = 10.21, *p* = 0.01, η_p_^2^ = 0.51). Importantly, the effect was significantly reduced for the 8-distractor displays, confirmed by a significant interaction of trial type and distractor set size (F(1,10) = 26.01, *p* < 0.001, η_p_^2^ = 0.72); see Fig. 7. There was no significant main effect of SOA (F(1,10) = 2.85, *p* = 0.12, η_p_^2^ = 0.22).

Figure 7 revealed a clear increase in mean saccadic reaction times with the singleton distractor trials, which produced a statistical reliable main effect across trial types (F(1,10) = 10.21, *p* = 0.01, η_p_^2^ = 0.51).

### Discussion

Experiments 1–4 have highlighted that the IRD effect is only present when a singleton distractor is utilised at short SOAs (400 ms), but not at long SOAs (2400 ms), and that the effect is reliant on successful inhibition of the distractor during search display_1_. Experiment 5 sought to explore the proposition that if inhibition of a distractor is required for the IRD effect, then the effect should still be present in the absence of a target in search display_1_. The present data supports this interpretation. Here we replicated the singleton distractor effect and show that there is no effect with multiple distractors. Importantly, the data demonstrated that a target is not essential to the IRD effect, which also differentiates it from negative priming tasks. Moreover, experiment 5 also highlighted that an overt eye movement during search display_1_ is not critical to the IRD effect.

## General discussion

The aim of this paper was twofold: firstly, to replicate the Inhibition of a Recent Distractor as reported by Crawford et al. (2005a), and secondly, to investigate various factors that may shed light on why evidence of saccadic inhibition is not observed when multiple distractors are presented. Experiment 1 successfully replicated the delay in the initiation of saccadic eye movements when a target appeared in the location of a recent distractor. Experiments 2 and 3 therefore, explored whether this IRD effect was the due to spatial or temporal variables. As these factors did not have a significant impact, experiments 4 and 5 explored how the importance of generating a saccade in the search display_1_ for Inhibition of a Recent Distractor (IRD).

The combined data presented across all experiments did not resolve all the issues, however, they do allow us to rule out several candidate hypotheses. The analyses presented in experiment 4 confirmed that the effect is dependent on successful inhibition of a saccade to the distractor. For a successful trial participants needed to effectively inhibit an eye movement towards the distractor; the IRD effect was absent on trials where participants did not first successfully inhibit a potentially erroneous saccade to the distractor. Experiments 1–5 also confirmed that the IRD effect was not detected when multiple distractors were presented; the effect was only evident when a single distractor was presented. An intriguing finding has emerged from research comparing the effects of single versus multiple distractors on the centre-of-gravity (or global effect) of saccadic landing positions, indicating that the disruptive impact of the distractor is primarily evident in the single distractor condition (McSorley and Findlay 2003). This phenomenon could provide insight into why inhibiting the distractor is particularly critical in single distractor displays. It is worth noting that in such displays, the distractor stands out as novel, distinctive, and highly conspicuous, unlike the homogeneity in the multiple distractors displays. This observation suggests that the saliency of the distractor may play a pivotal role.

Experiments 2 and 3 revealed that the IRD effect is not obscured by simple spatial or temporal factors. The presence of the distractor effect is not simply constrained or governed by the proximity of the distractor. Experiment 3 replicated the effect when a single distractor was present (although there was no significant impact of stimulus onset asynchrony). No effect was observed with multiple distractors, regardless of whether the delays were long (2400 ms) or short (400 ms). The last two experiments investigated the involvement of eye movements in the initial search display and verified that the IRD effect occurred solely on trials where an erroneous saccade to the distractor was not initiated. However, a saccade towards the target is not an essential requirement for the IRD effect. This suggests that a pivotal factor is the competition between the distractor and the target, but a saccade is not essential.

A primary strength to using the IRD task is that it addresses some of the challenges presented in the anti-saccade task. Unlike the anti-saccade task, the IRD task offers a target for directing the eye towards, which is in line with typical gaze behaviour in everyday situations. The IRD examines inhibition by probing the spatial effect of the previous distractor on the latency of the current saccade towards the subsequent target at that location. It does not mislead the participant about the future location of the target or requires an eye movement away from the target or cue. Instead, they are presented with two visual displays; the first being a target and distractor, followed by second display with a single target that varies in location. In the IRD task, inhibition is measured implicitly by contrasting the reaction times to the new location in relation to the distractor location in the previous display. This allows for a dual assessment of the facilitation of eye movements directed towards the target, and inhibition of eye movements towards a distractor (Crawford et al. 2005a). This more naturalistic assessment of inhibitory control may provide a different perspective when exploring capability among neurodivergent groups.

Impairments in inhibitory control have been reported across a series of different neurodevelopmental conditions: ADHD (Maron et al., 2022), dyslexia (Wilcockson et al. 2019), neurodegenerative: Alzheimer’s disease (Boxer et al. 2012; Crawford et al. 2005b; Crawford et al. 2019; Heuer et al. 2013; Kaufman et al. 2012; Molitor et al. 2015; Wilcockson, Mardanbegi, Xia, et al., 2019, Opwonya et al. 2022 for review), and other clinical diseases and disorders: Parkinson’s disease (Waldthaler et al. 2021 for review; Das et al. 2022). Given that the distractors affect the efficiency of goal-directed saccades, it would therefore be adaptive to mitigate their influence on gaze control. However, the deficits observed across a range of different clinical disorders in the AST, are not replicated in the IRD task. For example, Polden and Crawford (2021) utilised the IRD paradigm in an experiment with dementia patients, individuals with mild cognitive impairment and controls, illustrating that the inhibition of a distractor is preserved in people with early and chronic AD (Alzheimer s Disease). This preservation of inhibitory control in the IRD could prove to be a useful clinical tool.

So why might there be different inhibitory effects across these two paradigms? Despite both exploring inhibitory control, it cannot be assumed that both paradigms target the same control mechanisms. There are some key differences that are likely the driving factors behind the distinct inhibitory mechanisms deployed. The AST requires a motor signal to direct the eyes to the opposite location rather than a signal to suppress the target. In the IRD task, the anti-saccade, requiring the participant to direct their gaze away from the target, is absent. A competing distractor has been shown to be vital in generating the distractor inhibition in the IRD task (Donovan et al. 2012), which is absent in the anti-saccade task. Studies have shown that this is distinct from general gaze aversion which is present in the anti-saccade task. Crawford et al. (2005a) demonstrated that the anti-saccade is unable to generate the spatial inhibition at the location of a distractor which is found in the IRD task. Donovan et al. (2012) highlighted the importance of the distractor in the display as spatial inhibition is enhanced when a competing target is present. Given the importance of understanding the nature of deficiencies in inhibitory control, and the selective preservation of cognition in neurodivergent groups, these findings provide a new way of exploring inhibitory control in a naturalistic task.

### The potential role of Distractor Saliency

In our study, we utilized multiple distractors displays consisting of identical green disks alongside a singleton red target. These displays are known to induce a pop-out effect, where the target easily stands out from the background items (Maljkovic and Nakayama 1994). Furthermore, in such displays, the detection of the target remains unaffected by the number of similar background items, as they can be readily grouped and filtered from attention (Duncan and Humphreys 1989). This grouping process is automatic and imposes a low demand on attentional resources. This suggests that active inhibition plays a more significant role in displays where the non-target distractors are not homogeneous. According to the Duncan and Humphrey model, such displays result in competitive interactions among the background items. In contrast, in the singleton distractor display, there is a singleton salient distractor.

The P_D_ ERP, an electrical component marker of distractor inhibition with a range of 100–400 ms (Sawaki et al., 2012), has been used as evidence for distractor inhibition. Drisdelle and Eimer (2023) found evidence of distractor inhibition using high and low salient distractors when presented against a homogeneous distractor. ERP evidence for the inhibition of a salient distractor was also reported by Stillwell et al. (2022). The absence of inhibition for the multiple display is likely due to various factors related to the target displays, including distractor homogeneity and saliency. This should be explored in future work.

### Neural mechanisms of inhibition

The superior colliculus and frontal eye fields play crucial roles in the oculomotor network, mediating the competition between potential goals for saccadic eye movements. Targets and distractors generate corresponding and parallel activation of spatially distinct populations of neuronal activity. Various models of saccade generation propose that the coordination of eye movements, in terms of timing and direction, emerges from lateral neuronal interactions within the intermediate layers of the superior colliculus. These interactions integrate external and internal inputs within a retinotopically organized map (Godijn and Theeuwes 2002; Trappenberg et al. 2001; van Opstal and van Gisbergen 1989). The inhibition of a recent distractor observed in our study may reflect the long-range mutual inhibition between simultaneously activated colliculus sites in response to targets and distractors. A characteristic feature of the activation induced by a target and distractor is the saccadic initial deviation towards the distractor before redirecting towards the target (McPeek and Keller, 2001). This suggests that saccade execution may commence prior to the final selection of the saccadic goal. The initial curvature towards the distractor corresponds to presaccadic activity in the superior colliculus, indicative of distractor population activity. This activity is temporarily suppressed at the time of saccade initiation, while target-related activity persists (McPeek et al. 2003). However, the dynamics in the presence of multiple distractors remains unclear.

Walker et al. (1997) described a critical zone around the target, where distractor signals are integrated, resulting in saccades landing between the target and distractor (an averaging effect). If the distractor falls outside this zone, extended fixation units are activated, delaying the saccade to the target. This delay is associated with the widespread impact of spatial encoding dispersed throughout the superior colliculus, where peaks in the neuronal overlap, initiating saccades towards intermediate spatial locations. The increased delay to saccade towards the T-D trials in our task may reflect the activation of the fixation units responding to the previous distractor location. This suggests that fixation units can be activated by the memory location of a previous distractor, in a similar fashion to the account of inhibition of return suggested by Findlay and Walker (1999). However, multiple distractors have a lesser impact on saccadic landing positions compared to a single distractor (McSorley and Findlay 2003). Further research is needed to understand the processing of multiple distractors on current and future saccadic eye movements.

### Concluding remarks and future directions

The present experiments are important in that they rule out a series of potential explanations in relation to the IRD effect, but further experiments are required to explore other possibilities. For example, it has been proposed that salient objects have high potential to disrupt target performance, and as such participants learn to proactively suppress them (Gaspar et al. 2016). However, more recent research (Hauck et al. 2023) has proposed a more nuanced interpretation of the impact saliency has on inhibition. Future work should therefore expand on the idea of target and distractor saliency playing a significant role in the inhibition of a recent distractor effect.

There is increasing interest in the relationship of overt and covert aspects of visual attention. Compelling evidence indicates a reciprocal interaction between brain mechanisms primarily dedicated to coding visual stimuli and those primarily involved in eye movement (e.g. Moore and Armstrong 2003). Experiment 5 together with previous work confirms that IRD can be generated by covert attention as well as saccadic eye movements. Given that saccadic eye movements are associated with enhanced visual selection Casteau and Smith (2019), we suggest that attentional selection (either covert or overt) may be a key factor in the generation of IRD.

## Data Availability

Data for this study are available will be made available at the Lancaster University open access repository.
